# Supplementary material for: Dose-Dependent Blood-Feeding Activity and Ovarian Alterations to PM2.5 in Aedes aegypti
Source: Insects. 2021 Oct 18;12(10):948. doi: 10.3390/insects12100948 (PMC8539269; doi:10.3390/insects12100948)
Supplement: Supplementary file 1 [file insects-12-00948-s001.zip › insects-1390412-supplementary_Final.pdf]

Supplementary data

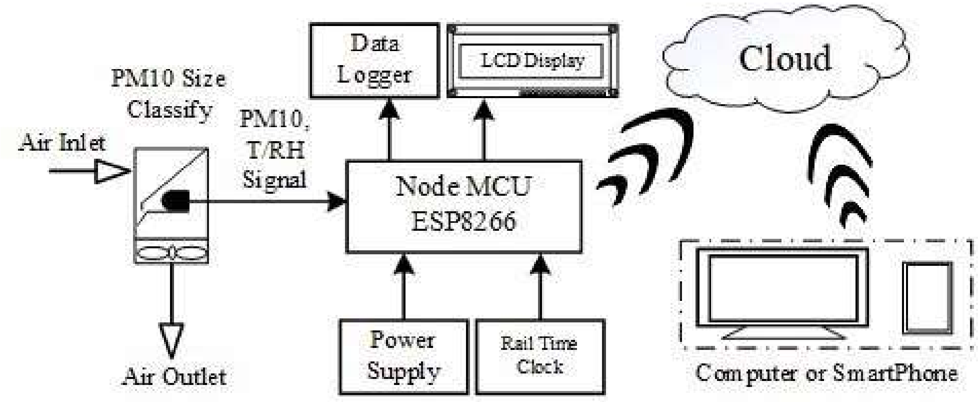

Figure S1. Operating Principle of DustBoy. Microcontroller unit; LCD liquid crystal display / RH&T Relative humidity & Temperature / RTC real time clock [27].

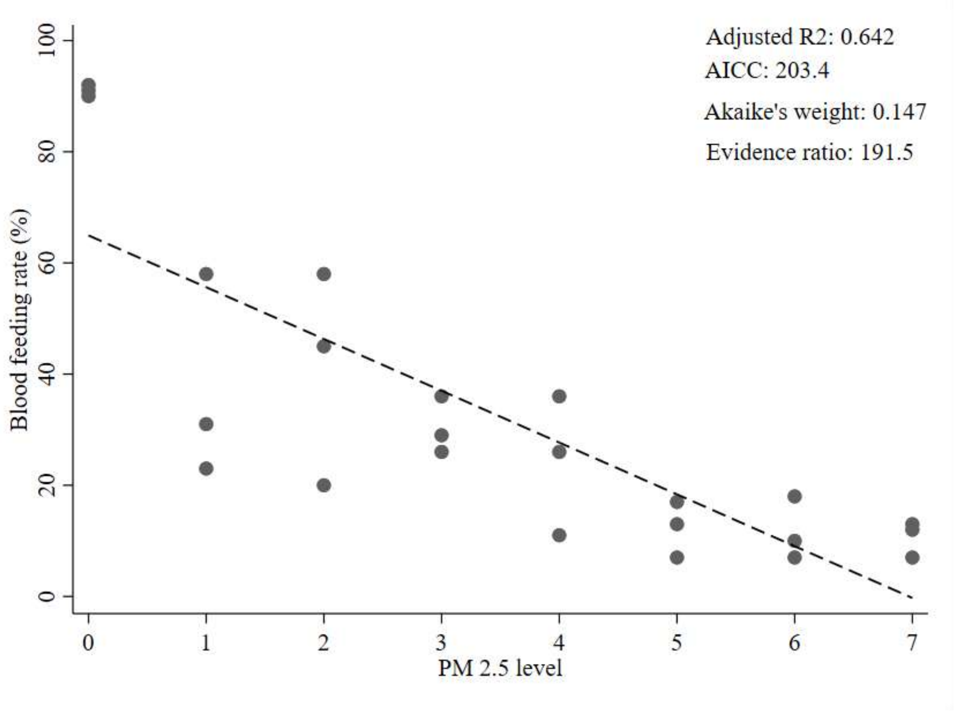

Figure S2. Linear model of the dose-response relationship between $PM_{2.5}$ level and blood-feeding rate in female *Ae.aegypti* mosquitoes

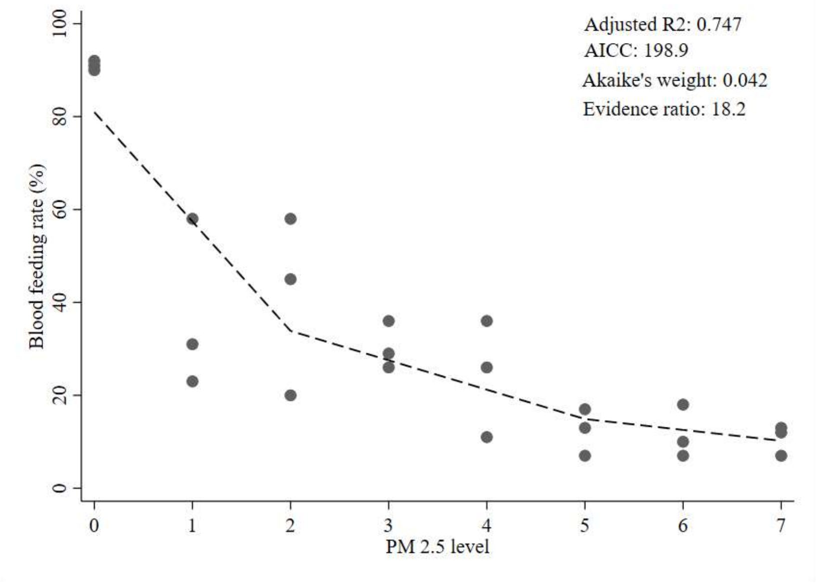

Figure S3. Three-segment piecewise linear of the dose-response relationship between $PM_{2.5}$ level and blood-feeding rate in female *Ae.aegypti* mosquitoes

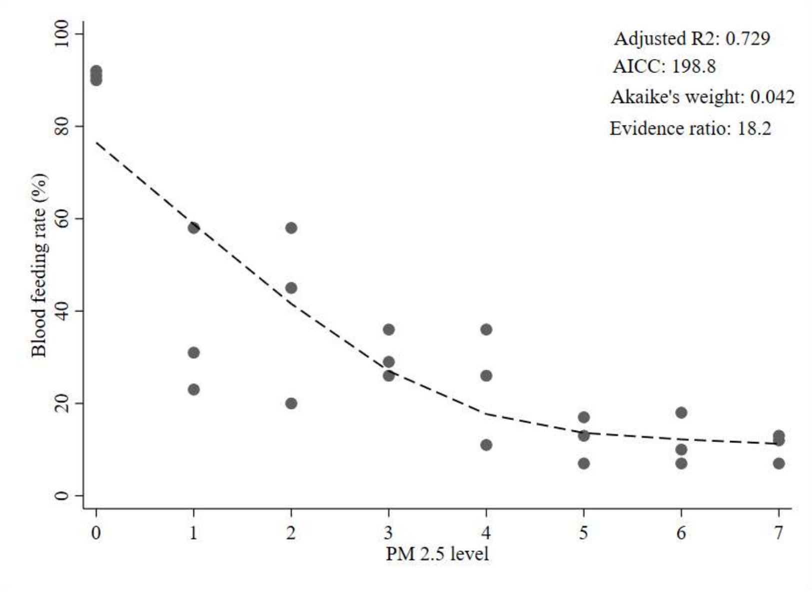

Figure S4. Three-knot restricted cubic spline model of the dose-response relationship between $PM_{2.5}$ level and blood-feeding rate in female *Ae.aegypti* mosquitoes

Table S1. Trace metal concentrations on the $PM_{2.5}$ filter paper

Metals	Concentration (mg/g)
**Iron (Fe)**	0.513 ± 0.112
**Zinc (Zn)**	0.087 ± 0.019
**Copper (Cu)**	0.015 ± 0.003
**Manganese (Mn)**	0.010 ± 0.002
**Nickel (Ni)**	0.015 ± 0.032
**Chromium (Cr)**	0.080 ± 0.017
**Cadmium (Cd)**	0.007 ± 0.002
**Lead (Pb)**	0.075±0.009

Notes:- Concentration is presented as mean ± standard deviation
